# Pericardial-Drain-to-Central-Venous-Line Return to Rescue Venoarterial Extracorporeal Membrane Oxygenation Flow in Tamponade From Stanford Type A Dissection: A Case Report

**DOI:** 10.7759/cureus.102350

**Published:** 2026-01-26

**Authors:** Gen Nakamura, Taichi Kato, Ken Inoue, Keita Shibahashi, Kazuhiro Sugiyama

**Affiliations:** 1 Tertiary Emergency Medical Center, Tokyo Metropolitan Bokutoh Hospital, Tokyo, JPN

**Keywords:** cardiac tamponade, central venous line, ecmo, pericardial drain, stanford type a aortic dissection

## Abstract

Cardiac tamponade secondary to Stanford type A aortic dissection carries extremely high mortality, and pericardiocentesis may precipitate catastrophic hemorrhage; during extracorporeal cardiopulmonary resuscitation (ECPR), elevated pericardial pressure can further impair venous return and limit venoarterial extracorporeal membrane oxygenation (VA-ECMO) flow. An 87-year-old woman suffered cardiac arrest due to tamponade from Stanford type A dissection and underwent ECPR, but venous drainage was severely compromised, and ECMO flow remained low (1.5-2.0 L/minute). Echocardiography showed progressive pericardial effusion, and pericardial drainage produced high-pressure bloody output consistent with ongoing rupture. As a rescue maneuver to maintain systemic perfusion, the pericardial drain was connected to a central venous line via an interposed syringe to reinfuse pericardial effluent into the venous circulation, improving ECMO flow to 2.5-2.8 L/minute and stabilizing hemodynamics long enough to proceed to surgical repair; computed tomography confirmed Stanford type A dissection. The patient underwent a bio-Bentall procedure but died of refractory coagulopathy and bleeding on postoperative day 2. Reinfusion of pericardial effluent into the central venous system may transiently restore venous return and stabilize ECMO flow in tamponade complicating Stanford type A dissection during ECPR and should be considered only as a high-risk, last-resort bridge under continuous hemodynamic and circuit monitoring until definitive surgical correction is achieved

## Introduction

Stanford type A aortic dissection can rapidly lead to circulatory collapse and, when complicated by cardiac tamponade, is associated with exceptionally high mortality rates [1]. Pericardiocentesis is the first-line intervention for cardiac tamponade, intended to relieve mechanical obstruction and restore cardiac output. However, in tamponade secondary to aortic dissection, pericardial drainage carries the risk of catastrophic hemorrhage due to rupture of the adventitia, potentially resulting in rapid exsanguination and failure to bridge the patient to surgery [2]. In such circumstances, extracorporeal cardiopulmonary resuscitation (ECPR) may serve as a salvage strategy to maintain circulation until definitive repair.

In cardiac tamponade, elevated pericardial pressure restricts cardiac filling and may also collapse the great veins, thereby reducing venous return. As a result, effective venous drainage to the ECMO cannula can be severely compromised despite adequate pump speed, leading to insufficient venoarterial extracorporeal membrane oxygenation (VA-ECMO) flow [3].

We report the case of a patient with Stanford type A aortic dissection who developed refractory venous drainage failure after ECPR initiation. Circulation was maintained by a novel maneuver in which pericardial effluent was reinfused through a central venous line, preserving VA-ECMO flow until surgery.

## Case presentation

An 87-year-old woman with a history of hypertension, postoperative endometrial cancer, primary hyperparathyroidism, monoclonal gammopathy, and chronic kidney disease was admitted to the emergency department. Her long-term medications included benidipine 4 mg once daily, azelnidipine 8 mg once daily, celecoxib 100 mg twice daily, and irsogladine maleate 2 mg twice daily. At baseline, she lived at home and was independent in most activities of daily living, requiring only minimal assistance.

She complained of headache and malaise since the morning and was found by family members in the evening to have collapsed in a crouched position on the toilet, prompting the activation of emergency medical services.

On prehospital assessment, she was noted to have impaired consciousness and respiratory failure: Glasgow Coma Scale (GCS) 7 (E1V2M4), pupils 2 mm bilaterally, respiratory rate (RR) 18 breaths/minute, heart rate (HR) 96 beats/minute, and unrecordable non-invasive blood pressure, although the radial pulse was palpable. With oxygen at 10 L/minute via a reservoir mask, her SpO₂ was 70%, and her body temperature was 35.4 °C.

**Figure 1:**
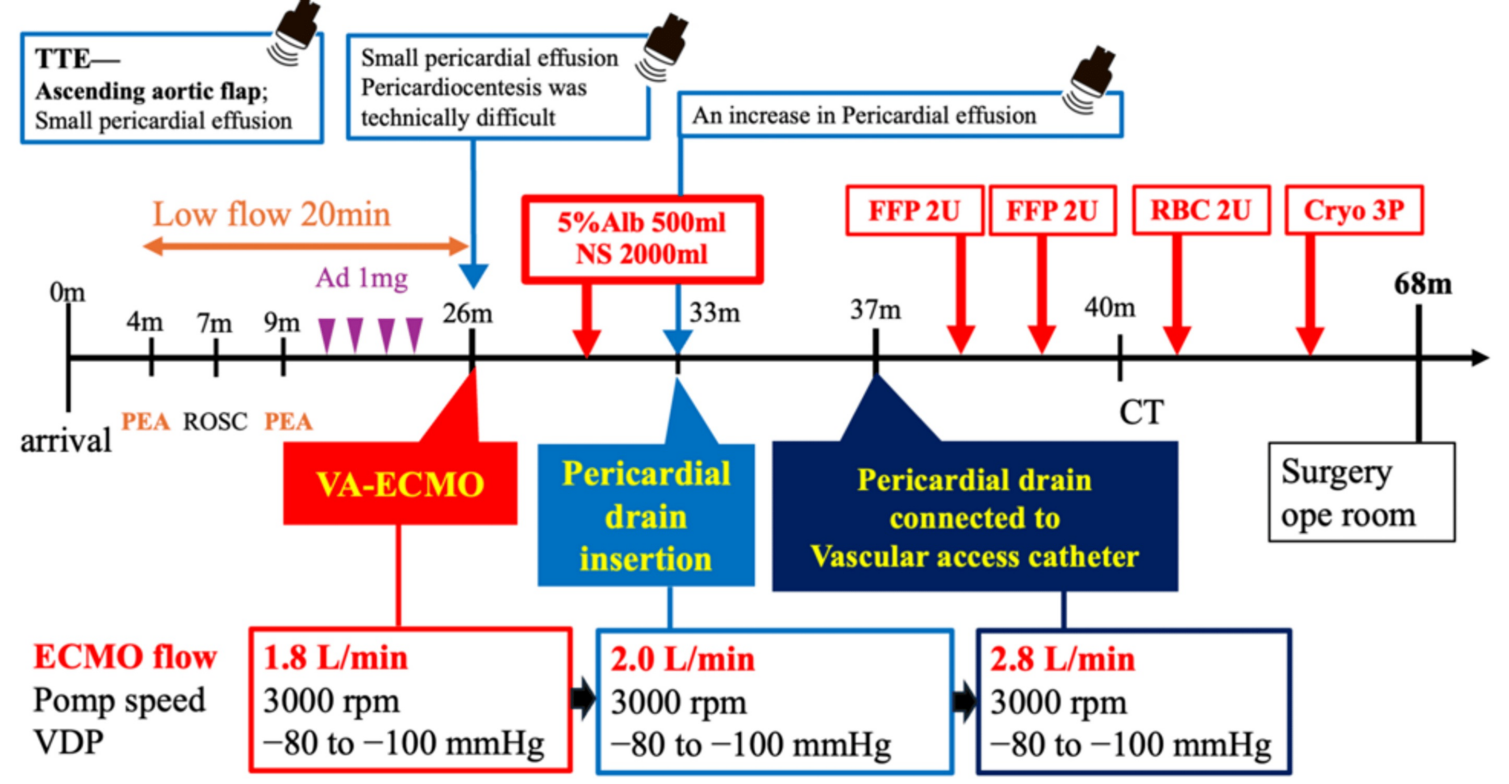
Timeline from symptom onset to transfer to the operating room. Image credit: All authors. Ad, adrenaline; PEA, pulseless electrical activity; ROSC, return of spontaneous circulation; VA-ECMO, venoarterial extracorporeal membrane oxygenation; NS, normal saline; TTE, transthoracic echocardiography; VDP, venous drainage pressure

Upon arrival at our hospital, her level of consciousness had deteriorated to a GCS score of 3 (E1V1M1), with 4-mm pupils bilaterally and sluggish light reflexes. She was in severe respiratory distress (RR 10 breaths/minute, HR 84 beats/minute, unmeasurable blood pressure, and SpO₂ 73% on 10 L/minute of oxygen via a bag-valve mask). Point-of-care ultrasonography revealed pericardial effusion approximately 1 cm posterior to the left ventricle and an intimal flap in the ascending aorta, suggesting cardiac tamponade secondary to a Stanford type A aortic dissection (Figure 1).

Given that the pericardial fluid was localized behind the left ventricular posterior wall, initial pericardiocentesis was attempted but was unsuccessful due to technical difficulties. Immediately after arrival, the radial pulse became impalpable, and pulseless electrical activity (PEA) ensued; return of spontaneous circulation (ROSC) was achieved after one cycle of chest compressions. After initial stabilization, the emergency physicians and cardiovascular surgeons discussed the prognosis and treatment options with the patient’s family. The shared decision was to pursue full escalation and continue invasive life-sustaining treatment. An arterial line was inserted for invasive blood pressure monitoring, and vasoactive agents were administered; however, she again progressed to PEA arrest. When the patient progressed to cardiac arrest, pericardiocentesis was attempted; however, within the echocardiographically visualized field, the pericardial effusion was shallow, and safe needle insertion during ongoing chest compressions was considered difficult. Although a surgical pericardial window was also considered, acute aortic dissection was strongly suspected as the primary pathology. Under these circumstances, opening the pericardium without access to an autologous blood recovery system was deemed to carry a high risk of uncontrollable hemorrhage due to potential rupture of the Valsalva sinus. Therefore, ECPR was selected. ECPR was initiated, and VA-ECMO was established using a 22-Fr drainage cannula in the right femoral vein and a 16-Fr return cannula in the right femoral artery. The ECMO circuit consisted of a centrifugal pump (HCF-MP23H) and a hollow-fiber HPO-23WH-C oxygenator (MERA CPB Circuit, Senko Medical Instrument Corp., Tokyo, Japan). The low-flow duration was 20 minutes.

Initial ECMO flow was limited to 1.5-2.0 L/minute because of impaired venous drainage. Increasing the pump speed beyond this range led to excessive negative drainage pressures (−80 to −100 mmHg) with marked venous line chattering, preventing further augmentation of ECMO flow. Despite fluid resuscitation, adequate blood flow was not maintained. Repeat ultrasonography revealed a marked increase in pericardial effusion, prompting pericardial drainage. Pericardial drainage was performed via a subxiphoid approach using a pericardial drainage catheter (Argyle Fukuroi Aspiration Seldinger Kit, Cardinal Health, Dublin, OH). The effluent was persistently bloody and under high pressure, sufficient to push the syringe plunger back.

**Figure 2:**
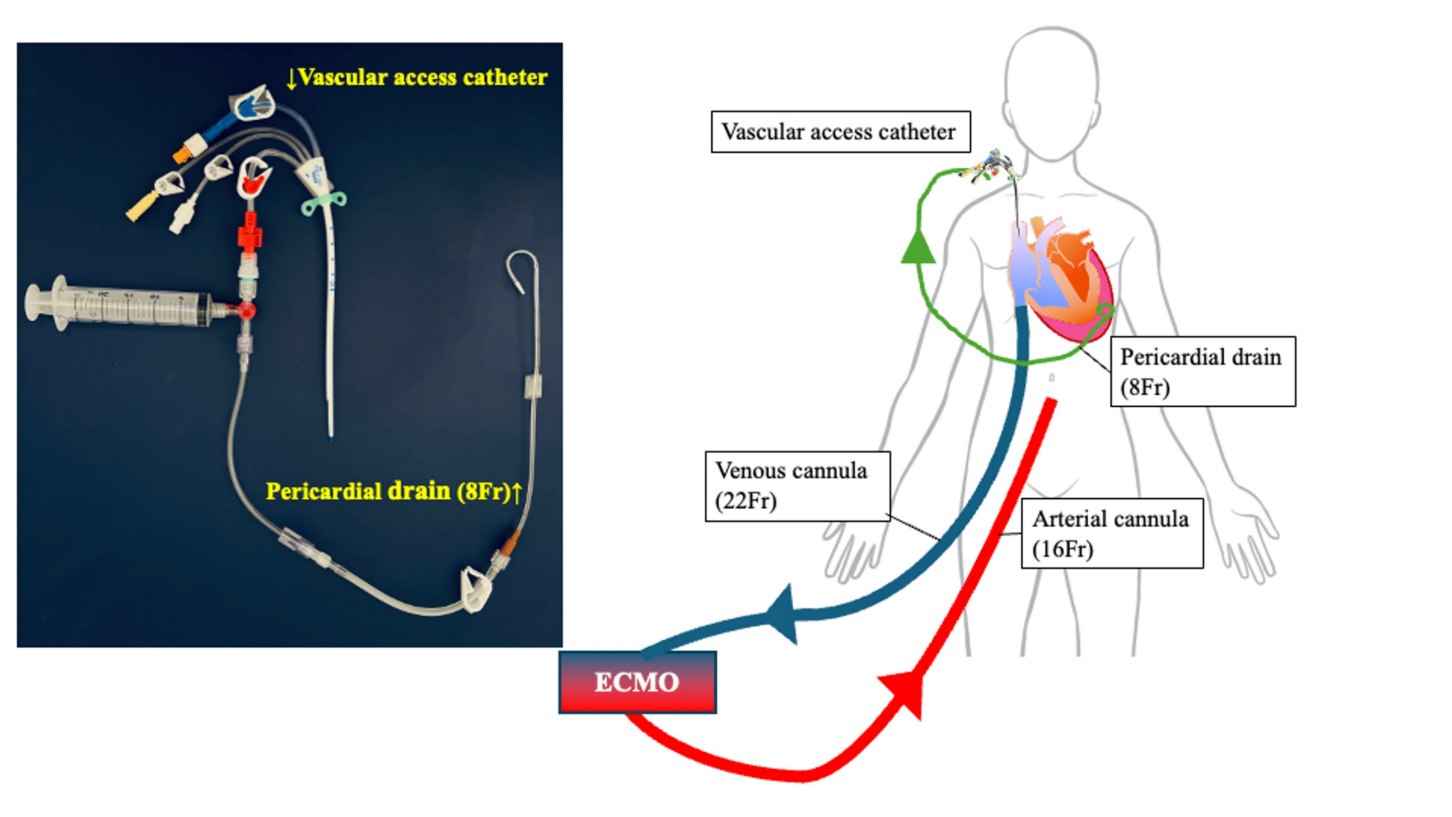
Schematic illustration of device connections among the ECMO circuit, pericardial drain, and vascular access catheter. The circuit consisted of a three-way stopcock, a locking 20-mL syringe, and a high-pressure extension tube (15 cm, male-to-male; Edwards; MK00589). No one-way valve was used. Image credit: Adapted from Kato et al. [4], with permission. ECMO, extracorporeal membrane oxygenation

**Figure 3:**
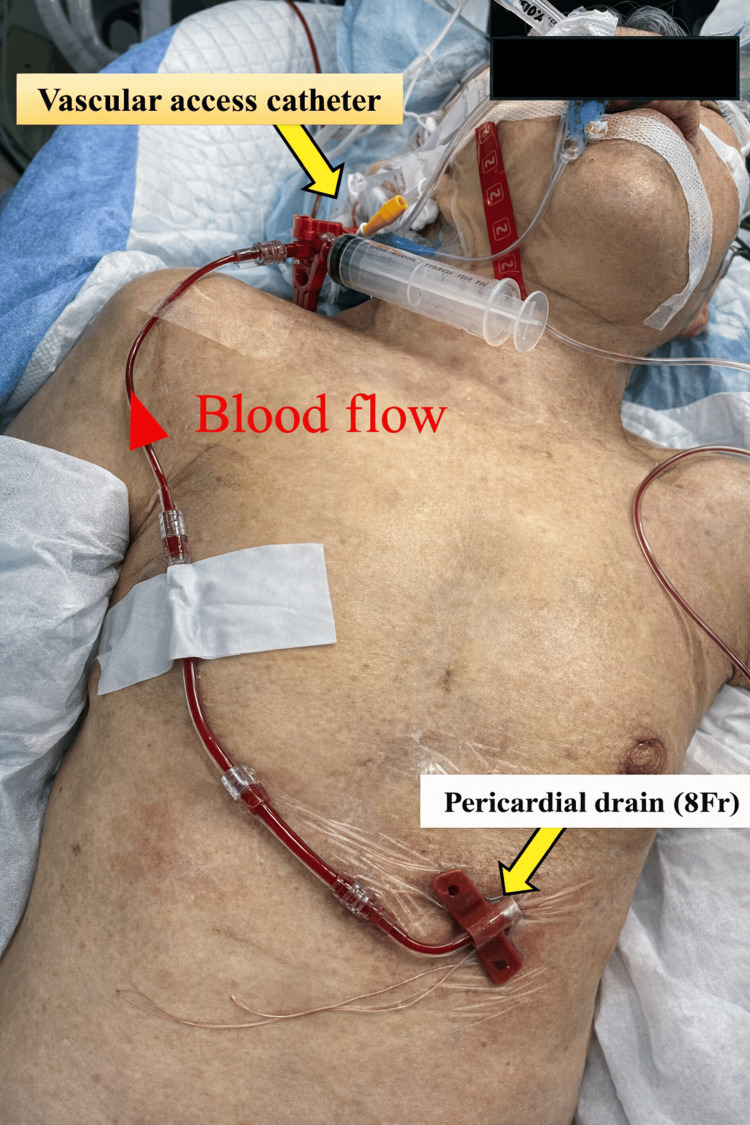
Bedside view illustrating the actual configuration connecting the pericardial drainage catheter to the central venous line in the patient.

To secure large-bore access for the expected massive transfusion during aortic dissection surgery, a Blood Access UK-Catheter kit (Nipro Corporation, Osaka, Japan) was placed via the right internal jugular vein. Although the pericardial fluid was drained, the continuous copious bloody output indicated impending massive hemorrhage, and ECMO flow remained around 2.0 L/minute. Suspecting that elevated intrapericardial pressure impaired venous drainage, we connected the pericardial drain to the central venous line via an interposed syringe, thereby returning the pericardial effluent to the venous system (Figures 2-3).

**Figure 4:**
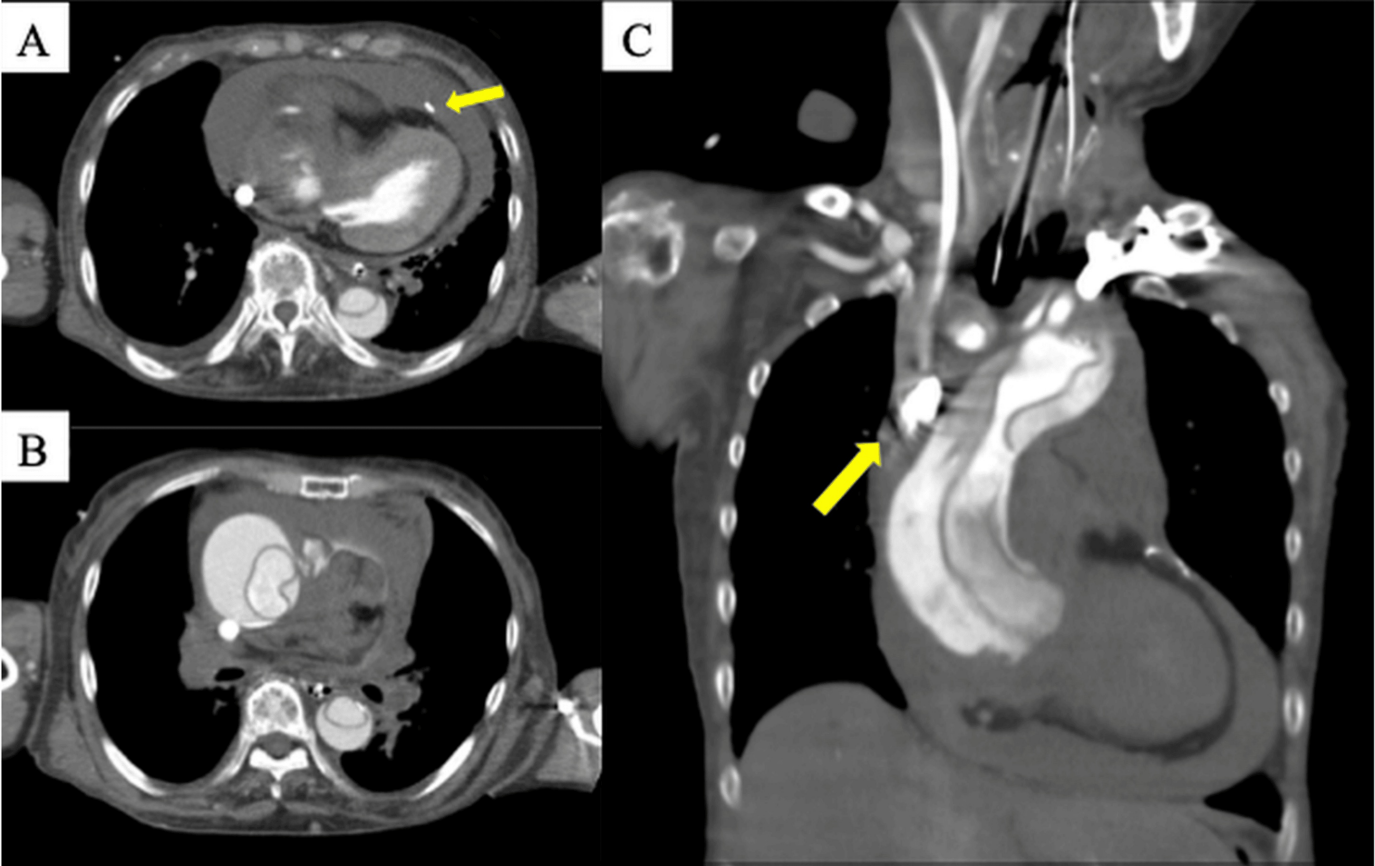
Contrast-enhanced computed tomography (CT) images. (A) Axial image at the right atrial level showing compression of the right atrium, with the drainage cannula positioned by the surrounding hematoma. The arrow indicates the pericardial drain. (B) Coronal image showing intimal rupture (entry) of the ascending aorta. (C) Axial image at the level of the superior vena cava (SVC) showing the drainage cannula advanced toward the SVC. The arrow indicates the tip of the ECMO drainage cannula in the SVC.

ECMO flow improved to 2.5-2.8 L/minute. Following stabilization of the patient’s condition with the aforementioned interventions, contrast-enhanced CT was obtained, confirming a Stanford type A aortic dissection (Figure 4), and the patient was transferred to the operating room 68 minutes after hospital arrival.

Surgical findings revealed adventitial disruption at the basal portion of the heart near the root of the right coronary artery following median sternotomy and opening of the pericardium, accompanied by active bleeding. The bio-Bentall procedure was performed on the left coronary artery, with Carrel-patch reconstruction and right coronary artery-saphenous vein graft bypass. The total operative time was 7 hours 35 minutes, cardiopulmonary bypass time was 6 hours 18 minutes, and aortic cross-clamp time was 4 hours 13 minutes. Owing to massive bleeding, the transfusion consisted of 28 units of red blood cells, 16 units of fresh frozen plasma, 40 units of platelets, and 9 units of cryoprecipitate. Postoperatively, persistent drainage insufficiency necessitated conversion to central ECMO, with drainage from the superior vena cava. Despite intensive care, coagulopathy and bleeding persisted, circulatory failure could not be reversed, and the patient died on hospital day 2.

## Discussion

This case involved cardiac arrest due to cardiac tamponade secondary to Stanford type A aortic dissection, in which venous drainage insufficiency persisted after ECPR. Although pericardiocentesis is the first-line treatment for cardiac tamponade, it was technically unfeasible in this patient because the effusion was located behind the left ventricular posterior wall. In tamponade caused by aortic dissection, pericardial drainage carries the risk of catastrophic hemorrhage due to rupture of the adventitia, often resulting in rapid re-accumulation of blood and loss of circulating volume. In such cases, establishing and maintaining perfusion until surgical repair becomes extremely challenging.

The standard management of Stanford type A aortic dissection consists of prompt analgesia and blood pressure control, followed by rapid transfer to the operating room for definitive surgical repair [5]. However, when circulation cannot be maintained preoperatively due to tamponade, pericardial drainage may be required [6]. The preoperative mortality of Stanford type A dissection complicated by tamponade is reported to be approximately 29%-32%, with outcomes even worse when cardiac arrest occurs [1]. Although the use of VA-ECMO for aortic dissection with profound circulatory failure or cardiac arrest remains controversial due to the potential risk of dissection propagation, it may serve as a temporary measure in cases where pericardiocentesis is unsuccessful or contraindicated [3,7,8]. In such settings, venous return may be markedly reduced because the elevated intrapericardial pressure collapses the right atrium and impairs venous drainage [9,10].

While pericardial drainage can decompress the pericardial space, the evacuated blood is often hemorrhagic and continuously reaccumulates owing to active extravasation from the dissected aortic root. This effluent risk is simply discarded, precipitating hemorrhagic shock. Previous reports have described connecting the pericardial drain directly to the ECMO drainage limb to auto-transfuse the effluent and maintain circuit flow. Kato et al. reported a case of post-myocardial infarction free-wall rupture in which connecting the pericardial drain to the drainage cannula restored flow during ECPR [4]. Ijuin et al. described a similar technique in a patient with a Stanford type A dissection and tamponade [11].

In our patient, we adopted a modified approach by connecting the pericardial drain to a central venous line rather than directly to the ECMO drainage limb. Compared with direct reinfusion into the drainage cannula, this configuration likely attenuated the transmission of excessive negative pressure to the pericardial drain and reduced the risk of air entrainment into the ECMO circuit. In addition, avoiding direct connection to the drainage limb may have limited the potential for clot propagation into the ECMO circuit in the setting of ongoing hemorrhagic pericardial effusion. With this modified configuration, partial autotransfusion of the effluent was achieved, and VA-ECMO flow improved from 1.5-2.0 to 2.5-2.8 L/minute, allowing maintenance of systemic perfusion until definitive surgical repair.

Although re-transfusion of shed blood may theoretically exacerbate coagulopathy, this patient already exhibited markedly elevated D-dimer and fibrin degradation product levels at presentation, suggesting that consumptive coagulopathy was established before re-transfusion. In desperate situations where operative entry is delayed and circulatory collapse is imminent, temporary reinfusion of pericardial effluent through a central venous line may serve as a life-saving bridge to surgery, provided the risks are carefully weighed.

In our experience, temporarily returning the pericardial effluent to the central venous line stabilizes the ECMO flow and systemic perfusion, enabling safe transfer to the operating room. This maneuver can be viewed as a physiological bridge that maintains the preload and venous return while decompressing the pericardial space. However, this strategy is inherently hazardous and should only be performed under continuous hemodynamic and circuit-monitoring conditions.

Future reports and experimental models are needed to better define the hemodynamic effects and safety of such *autotransfusion-like* techniques. In resource-limited or time-critical situations in which surgical entry is delayed and pericardiocentesis is not feasible, this approach may serve as a temporary life-saving bridge, buying time for definitive repair.

## Conclusions

In this single case, temporary reinfusion of pericardial effluent through a central venous line was associated with stabilization of ECMO flow and systemic perfusion in a patient with Stanford type A aortic dissection complicated by cardiac tamponade, when pericardiocentesis was not feasible, or drainage was insufficient. Although inherently risky and based on an inferred physiological mechanism, this maneuver may represent a short-term physiological bridge to definitive surgical repair. However, its generalizability remains uncertain, and further accumulation of similar cases is needed to better define its role and safety.

## References

[1] Gilon D, Mehta RH, Oh JK (2009). Characteristics and in-hospital outcomes of patients with cardiac tamponade complicating type A acute aortic dissection. Am J Cardiol.

[2] Hayashi T, Tsukube T, Yamashita T (2012). Impact of controlled pericardial drainage on critical cardiac tamponade with acute type A aortic dissection. Circulation.

[3] Yukawa T, Sugiyama K, Miyazaki K, Tanabe T, Ishikawa S, Hamabe Y (2018). Treatment of a patient with acute aortic dissection using extracorporeal cardiopulmonary resuscitation after an out-of-hospital cardiac arrest: a case report. Acute Med Surg.

[4] Kato T, Miyagawa A, Hikone M, Yuri K, Sugiyama K (2023). Peripheral VA-ECMO and pericardial drainage connected to the ECMO circuit for cardiac tamponade from blowout rupture: a case report. BMC Cardiovasc Disord.

[5] Erbel R, Aboyans V, Boileau C (2014). 2014 ESC Guidelines on the diagnosis and treatment of aortic diseases: Document covering acute and chronic aortic diseases of the thoracic and abdominal aorta of the adult. The Task Force for the Diagnosis and Treatment of Aortic Diseases of the European Society of Cardiology (ESC). Eur Heart J.

[6] Hiratzka LF, Bakris GL, Beckman JA (2010). 2010 ACCF/AHA/AATS/ACR/ASA/SCA/SCAI/SIR/STS/SVM guidelines for the diagnosis and management of patients with Thoracic Aortic Disease: a report of the American College of Cardiology Foundation/American Heart Association Task Force on Practice Guidelines, American Association for Thoracic Surgery, American College of Radiology, American Stroke Association, Society of Cardiovascular Anesthesiologists, Society for Cardiovascular Angiography and Interventions, Society of Interventional Radiology, Society of Thoracic Surgeons, and Society for Vascular Medicine. Circulation.

[7] Zhu G, Yue W, Li Y (2025). Successful resuscitation of acute type A aortic dissection with pulmonary embolism using long-term venoarterial extracorporeal membrane oxygenation: a case report. Front Med (Lausanne).

[8] Tsangaris A, Alexy T, Kalra R, Kosmopoulos M, Elliott A, Bartos JA, Yannopoulos D (2021). Overview of veno-arterial extracorporeal membrane oxygenation (VA-ECMO) support for the management of cardiogenic shock. Front Cardiovasc Med.

[9] Kondo T, Morimoto R, Yokoi T (2019). Hemodynamics of cardiac tamponade during extracorporeal membrane oxygenation support in a patient with fulminant myocarditis. J Cardiol Cases.

[10] Morcos M, Vincent L, Harari R, Badulak J, Chen M (2021). Cardiac tamponade in venoarterial extracorporeal membrane oxygenation. Echocardiography.

[11] Ijuin S, Takeuchi M, Nakai C (2021). Rescue pericardial drainage and return connected to ECMO for aortic rupture into the pericardial sac with acute type A aortic dissection. Case Rep Acute Med.

